# The optimal performance target of valuation adjustment mechanism agreement with real options perspective

**DOI:** 10.1371/journal.pone.0277509

**Published:** 2022-11-21

**Authors:** Yong Xue, Xinyi Yun

**Affiliations:** Department of Finance, School of Economics, Xi’an University of Finance and Economics, Xi’an, Shaanxi, China; URV: Universitat Rovira i Virgili, SPAIN

## Abstract

The valuation adjustment mechanism (‘VAM’) agreement has recently been widely adopted in venture capital investment in emerging markets. The VAM agreement endows venture capital institutions a contractual right to reevaluate invested startup contingent on preset performance targets, which is crucial for the effectiveness of the VAM agreement by deeply affecting the strategy and fate of the startup. Motivated by exploring a rational performance target setting, this paper: 1)Firstly, extracts a general structure of the VAM agreement by cases analysis;2) Secondly, adopts a real options methodology to derive the option value held by venture capital institutions and how the pre-determined performance target affect the payoff of venture capital institutions;3)Thirdly, derives the expected time to achieve the given performance target and the behavior choice of entrepreneurs of startups; 4)Finally, by maximizing the contractual value of venture capital institutions with the participation constraints of the entrepreneur, derives the optimal performance target setting. The result finds that the option value of the VAM agreement is positively related to the performance target. It may partially explain why venture capital institutions tend to dominate overly high targets from a real options perspective. We also confirm the incentive effect of the VAM agreement that entrepreneurs tend to exert their best effort. Furthermore, the derivation of the optimal performance target shows that it is an increased function of the agreement period and a subtractive function of project risk. This paper contributes to the literature on contingent payment mechanisms and provides practical implications for the VAM agreement design.

## Introduction

Since the low success rate of startups is primarily attributed to the shortage of funding [1], venture capital support has been illustrated as one of the main drivers for the growth of startups [2]. In addition to providing financial support to startups as the primary financing source [3–5], venture capital institutions also offer professional guidance for business management [6–8] and play an active role in promoting corporate innovation and cultivating entrepreneurship [9, 10], thereby helping startups to develop sustainably [11, 12]. Unlike bank lending, venture capital institutions do not care about the restriction of the limited tangible assets of the startups. Still, they pay more attention to the development potential, such as the management team’s ability and the enterprise’s core competitive advantage to carry out equity investment. The payoff of venture capital institutions mainly comes from the equity value of the invested startup, so careful selection and reasonable valuation of investment projects are crucial to the survival of the venture capital institutions [13, 14]. However, it is not easy for venture capital institutions to accurately evaluate the startup. On the one hand, the growth potential of startups is highly uncertain due to the influence of many factors such as policy, technology, and management in the early stage [15]. On the other hand, there is an agency problem due to information asymmetry [16, 17]. Therefore, Sahlman (1990) [18] pioneered those venture capital institutions should design a favorable investment contract rather than wasting time and energy evaluating the uncertain natural prospects of startups.

According to the basic contractual logic giving venture capital institutions both downside protection and a favorable position if the investee proves its potential [19], convertible security has been widely used as an investment mechanism in mature markets. Still, with the insufficiency of financial instruments such as convertible preferred stock in emerging markets, a contractual innovation—the valuation adjustment mechanism (‘VAM’) agreement—has been prevalent [20]. A VAM agreement, also known as a ‘Bet-on’ agreement, is a contingent payment arrangement concluded between venture capital institutions and portfolio startups upon predefined performance targets, following which venture capital institutions may exercise a contractual right to adjust the valuation [21–23]. When the startup cannot deliver the promised level of performance, it has to repay a specific value to venture capital institutions to compensate for their losses from over-evaluation; however, if performance targets are met, the startups may obtain an additional payment to offset their losses from under-evaluation. The clear advantage of adopting the VAM agreement is that it reconciles the differences between venture capital institutions and startups on the ex-ante understanding or belief of enterprise value when negotiating the transaction price and thus helps complete the investment process smoothly. The VAM agreement also creates a ‘shared vision’, which is argued as a mechanism that can alleviate disputes between investment and financing parties [24]. Moreover, it helps to mitigate the risk of adverse selection and moral hazard due to information asymmetries [25–28]. If venture capital institutions offer too low, the inherent high-quality startups may exit the market; however, if venture capital institutions offer too high, the startups may not work hard to deliver the promised performance. Furthermore, the punishment/award feature of the VAM agreement ensures a relatively fair transaction value, which inspires the invested startups or the management team to strive. Last, the VAM agreements may not only significantly affect the expected earnings of startups [29], but also convey positive signals to the market that the funded startup is confident with internal quality and prospects [22].

The VAM agreement offers a valuable mechanism to bridge the ex-ante valuation gap and align the startups’ interests with venture capital institutions. However, it has been documented excessive performance targets may lead to irrational operation of startups in pursuit of short-term benefits regardless of sustainable development [30–32]. A more critical question of how to determine a proper performance target is raised. To the best of our knowledge, relatively few studies have analyzed this issue. Lin (2020) [20] argues that the standards of success are likely to be determined by bargaining; thus, the final agreed performance target is more likely to represent a better bargaining position than the optimal common interests. In practical circumstances, where the startups can hardly raise financing from other channels, the venture capital institutions with more substantial bargaining power may impose an overly high-performance target, which may result in failures and defeats the purpose of venture financing. Song et al. (2019) [33] argue the agency problem may incur an overly high-performance target of VAM contract in acquisition-the acquirer’s top management team may use performance commitment to send a positive signal to the market to serve their self-interests such as cash bonus while neglecting whether the standards can be achieved and thus damage the effectiveness of performance commitment contracts. Zeng et al. (2020) [34]find the accounting conservatism of the acquirer to be positively associated with the target’s fulfillment of the performance commitments in VAM contracts; one reason is that the managers of conservative firms are more cautious, tending to lower the pre-specified performance standards to make them easier to fulfill and reduce the likelihood of asset impairment. Li and Li (2022) [35] find the acquirer’s CSR plays a positive role in the target’s fulfillment of performance commitment in the VAM agreement.

Prior studies have consented that performance target is crucial to the effectiveness of VAM agreement and found some attributes of investor, like bargaining power, agent’s self-interest, or conservatism, may affect the determination of performance target. However, two facts cannot be ignored for the rationality of the performance target. First, the effort of the invested startup is of the same significance in determining the performance target. Venture capital institutions may dominate the performance target, and the startup may have no choice but to accept an overly high standard. But if startups expect to fail to meet performance targets even with the best effort, the alternative option may be to reduce efforts to maximize their benefits. An excessively high target will undermine the incentive effect of the VAM agreement. Second, achieving performance targets is often subject to external factors such as macroeconomic and industry policy changes. When faced with changes in external conditions that may be positive or negative, there is a lack of intuitive understanding of how to set performance targets. To fill this research gap, this paper exploits the real options methodology to examine the economic value venture capital institutions acquire through the VAM agreement and startups’ behavioral choices to achieve their performance target. Assuming that venture capital institutions maximize their economic value within the constraint that startups expect to achieve their performance target, this paper derives an optimal performance target.

Our study makes three primary contributions. First, our work contributes to the literature on the VAM agreement and, more generally, the contingent payment mechanism. By exploiting the real options methodology, we formally derive the economic value of the VAM agreement, which is positively related to the pre-determined performance target. The result may provide a theoretical explanation for previously documented overly high-performance targets imposed by investors. By introducing startups’ behavioral choices to achieve performance targets, we confirm the VAM agreement’s incentive effect and provide new insights into how to set optimal performance goals. Second, our findings are of practical implications for VAM contract design. By maximizing the payoff of venture capital institutions while the startups expect to achieve their performance target, the derivation of the optimal performance target suggests that it should be positively correlated with the duration of the VAM agreement and negatively correlated with internal and external uncertainties. Third, we extract the general structure and execution sequence of the VAM agreement from the analysis of two classic cases, which may provide a reference for further theoretical modeling.

The rest of the paper is organized as follows. Section 2 analyzes the characteristics of the VAM agreement and extracts the general structure and execution sequence. Section 3 derives the economic value acquired by venture capital institutions through the VAM agreement and how that value relates to the pre-determined performance target. Section 4 analyzes the behavioral choices of startups and derives the optimal performance target. Finally, section 5 discusses limitations and future research directions, and section 6 concludes the paper.

## Characteristics and structure of the VAM agreement

### Cases of the VAM agreement

China has witnessed many investment events using the VAM agreement as the largest emerging market, including both win-win and failure cases. Thus, the VAM agreement is not only a simple investment tool but also profoundly impacts startups’ development strategy and destiny.

Since the specific form of the VAM agreement is flexible, this section takes the VAM agreement signed by Morgan Stanley and Mengniu in 2002 and the VAM agreement signed by Morgan Stanley and Yongle in 2005 as cases to extract the general characteristics and structure of the VAM agreement. Mengniu and Yongle both adopted the VAM agreement for venture capital financing, while Mengniu ended the deal with a win-win situation for both sides, but Yongle failed.

#### Mengniu’s VAM agreement

To introduce venture capital institutions to meet the financing needs of enterprises and create a flexible equity basis for an overseas listing, Mengniu first established the Cayman Islands company and Mauritius company. At the same time, Mengniu found Jinniu and Yinniu in the Virgin Islands. Among them, Jinniu shares are held by Mengniu executives, and Yinniu shares are held by senior executives related to the Mengniu business. Before the introduction of venture capital institutions, Jinniu and Yinniu each accounted for 50% of the shares of the Cayman Islands company. In the first round of capital injection, Jinniu and Yinniu purchased 1134 class A shares and 2968 class A shares of the company, accounting for 9.4% of the equity and 51% of the company’s voting rights. Morgan Stanley, CDH, and CDC have injected the US $25.97 million, holding 48980 class B shares, accounting for 90.6% of the equity and 49% of the voting rights. The funds injected by the venture capital institutions are used to purchase 66.7% of the registered capital of Mengniu shares.

**Fig 1:**
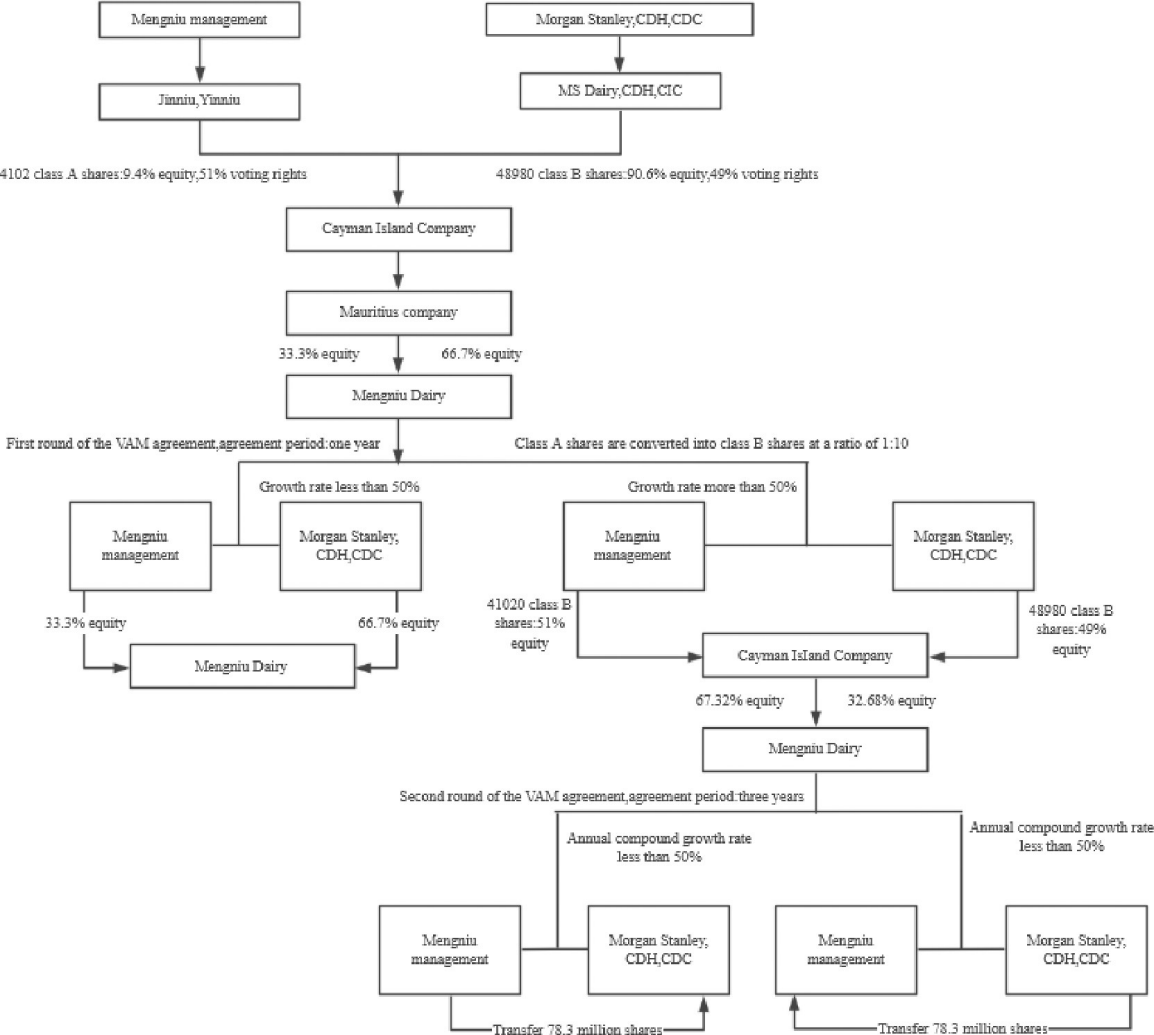
Mengniu’s VAM agreement.

After the first capital injection in 2002, venture capital institutions and Mengniu management signed a VAM agreement. The duration of the deal is one year. Suppose the agreed performance is achieved one year later. In that case, Mengniu management can convert the class A shares held by Mengniu into class B shares in the proportion of 1:10, accounting for 51% of the equity of the Cayman company, to achieve the consistency of equity and voting rights. If Mengniu’s performance fails to meet the target, venture capital institutions will obtain 60.4% of Mengniu’s absolute control. From 2002 to 2003, as Mengniu’s management achieved the performance target, the management accounted for 51% of the equity of the Cayman company, and the proportion of equity holding Mengniu Dairy increased to 67.32%. The ratio of equity containing Mengniu Dairy held by venture capital institutions was 32.68%. After the end of the first round of financing and agreement, in 2003, Morgan Stanley and other venture capital institutions purchased the convertible note the Cayman Islands company issued for the second round of funding with the US $35.23 million. The convertible note’s conversion price was only HK $0.74 per share, and signed the VAM agreement for three years. Since 2003, if the compound annual growth rate of Mengniu’s profit is not less than 50%, the venture capital institutions will transfer 78.3 million shares to the management of Mengniu. If the performance target is not achieved, the management of Mengniu will transfer the same amount of shares to the venture capital institutions. From 2003 to 2005, since the development of Mengniu far exceeded the performance target agreed in the agreement, the venture capital institutions paid the management of Mengniu a convertible note with the principal of US $5.98 million in 2005, which terminated the VAM agreement in advance, and the two sides achieved a win-win situation. Fig 1 shows the main contents of the VAM agreement signed by venture capital institutions and Mengniu.

#### Yongle’s VAM agreement

**Fig 2:**
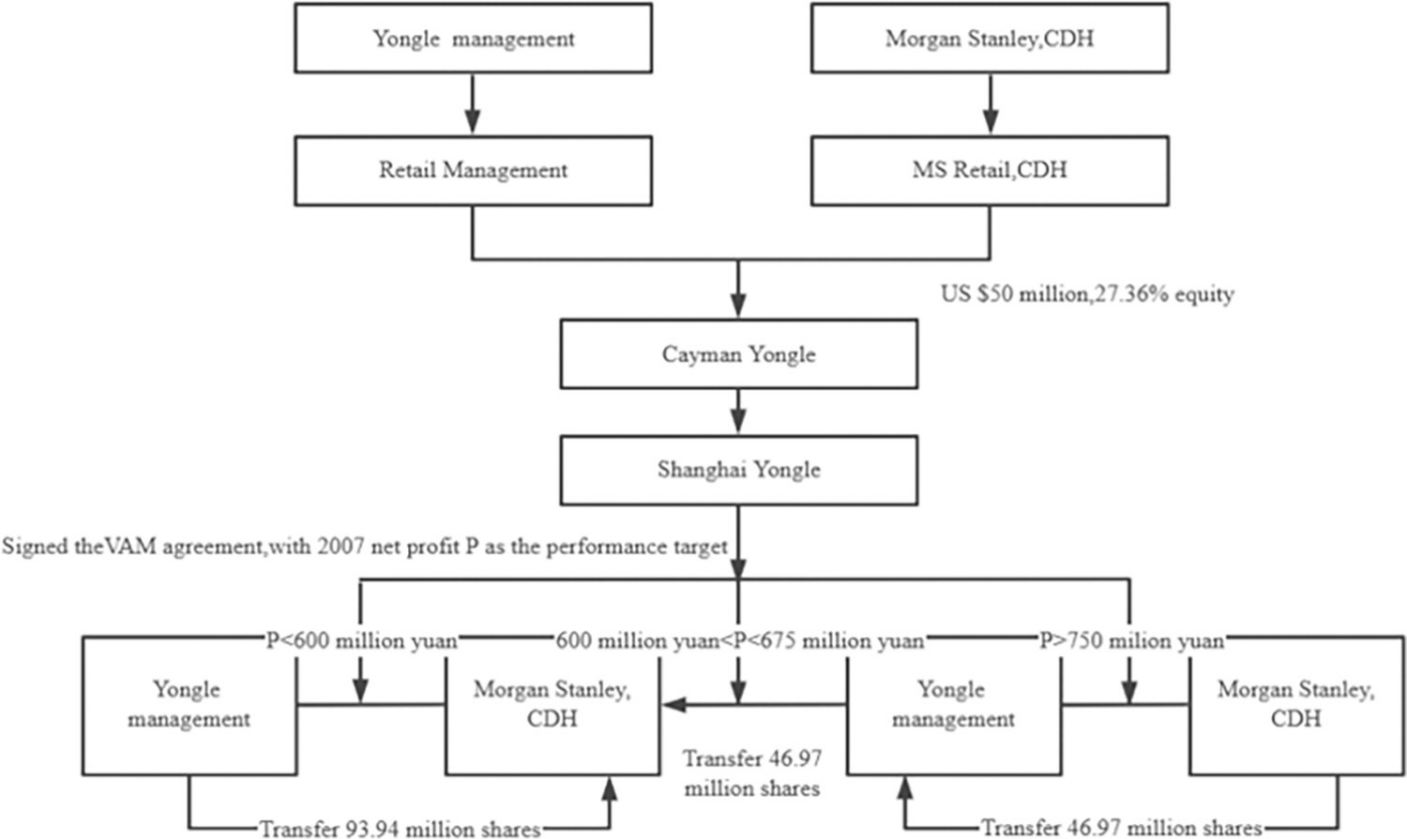
Yongle’s VAM agreement.

Like Mengniu, Yongle carried out overseas restructuring in 2004. First, it established Retail Management in the Virgin Islands, whose shareholders and the shareholding ratio was the same as those of Shanghai Yongle in China. Then, it found Yongle Electric Appliance Sales in the Cayman Islands, becoming the main body for introducing venture capital institutions and overseas listing. After that, in 2005, Morgan Stanley and CDH purchased 363 million shares and 59.13 million shares of Yongle through their subsidiaries, MS Retail, and CDH, obtaining 27.36% equity. As a result, the venture capital institution and Yongle management have signed a VAM agreement. If the profit of Yongle in 2007 is more than 750 million yuan, the venture capital institutions will transfer 46.97 million shares to Yongle management. If the profit is less than or equal to 675 million yuan, the Yongle management will transfer 46.97 million shares to the venture capital institutions. If the profit is not more than 600 million yuan, Yongle management transferred 93.94 million shares to venture capital institutions. After the introduction of venture capital institutions, China Yongle was listed in Hong Kong. A total of 518 million shares were issued in Hong Kong, with a financing amount of 1.2 billion yuan. Since the launch of the VAM agreement, Yongle has entered a stage of rapid mergers and acquisitions expansion. Because it is challenging to integrate mergers and acquisitions units in a short period effectively, the cost of mergers and acquisitions synergy is high, and the operating cost is much higher than that of competitors Gome and Suning. The decline in performance led to a sharp decrease in Yongle’s share price. As the agreement expired, Yongle, which had been listed for only nine months, finally chose to be merged by Gome, ending the VAM agreement. Fig 2 shows the main contents of the VAM agreement signed by venture capital institutions and Yongle.

### Characteristics of the VAM agreement

Based on the above facts, the characteristics of the VAM agreement can be summarized as follows:

First, the VAM agreements reconcile valuation differences between investors and investees. The financing parties involved in the VAM agreements are often startups in rapid development, which increase but lack cash flow. With narrow access to financing, high-growth startups introduce venture capital, reduce debt ratios, and gain value-added services from venture capital institutions in management, marketing, etc. When venture capital institutions inject money into startups and obtain equity, they first evaluate the project value and development prospects. However, the future development potential of the enterprise is full of uncertainty. In the early stage, the enterprise mainly faces product technology risk, management risk, and market demand risk. After successfully entering the growth period, the product enters the marketing stage, and the market share and future competition risk the profit. This makes it impossible for both sides of investment and financing to predict enterprises’ future development prospects. Although venture capital institutions can acquire specific information about enterprises through business plan screening, entrepreneur financing demonstration, due diligence, and other means, there is still a huge information asymmetry between venture capital institutions as financial investors and financing enterprises in professional fields, which also leads to differences in project evaluation between the two sides. The original intention of the VAM agreement was to reconcile the valuation differences between the investing and financing parties through the redistribution of rights afterward so that the investment process of venture capital institutions could proceed smoothly.

Second, there is the incentive effect of the VAM agreement. After the investment, venture capital institutions usually occupy a seat on the board of directors to participate in the decision-making of significant events and obtain business information. However, as financial investors, venture capital institutions do not intervene in daily business activities, which entrepreneurs and the original management usually control. Venture capital institutions mainly care about financial returns. In contrast, entrepreneurs care about financial returns and pay attention to private benefits of control in enterprise operation, including a sense of achievement, reputation, and on-the-job consumption. Because the interests of entrepreneurs and venture capital institutions are not precisely aligned, there may be moral hazard issues. For venture capital institutions, the return on investment ultimately depends on business performance. Therefore, it is necessary to restrict the moral hazard tendency of entrepreneurs and encourage their performance creation. As shown in the cases of Mengniu and Yongle, the VAM agreement stipulates that when the enterprise’s operating results exceed the preset performance objectives, equity incentives will be given to entrepreneurs. If the goals are not met, the management’s shares will need to be transferred to venture capital institutions to realize the transfer of control to a certain extent. Because entrepreneurs attach great importance to enterprise control through equity incentives and punishment measures, the interests of entrepreneurs can be bound with venture capital institutions.

Third, a reasonable performance target is crucial for achieving a win-win situation. The VAM agreement is an investment tool of an optional nature. Both the investment and financing parties bet on future uncertainties; the financing party exercises its rights if it meets certain trigger conditions, and the investor exercises its rights if the conditions are not met. Appropriate performance target setting can motivate entrepreneurs, but the pressure brought by the too high-performance target may make enterprises choose short-sighted and irrational business strategies. In the case of Yongle, the compound annual growth rate of Yongle’s profit from 2003 to 2005 is 40%. Even if this growth rate is maintained, the profit in 2007 will only reach 566 million yuan, far lower than the performance target of the equity award of 750 million yuan in the VAM agreement. The harsh performance target makes Yongle choose large-scale mergers and acquisitions expansion. Still, it is difficult to effectively integrate the mergers and acquisitions resources in the short term, increasing the management cost and reducing profitability. For venture capital institutions, the financial return ultimately depends on improving the enterprise’s fundamental profit level. Although the VAM agreement gives the venture capital institutions specific equity compensation when the performance does not reach the target, if the enterprise’s operation has been in trouble, it may also cause losses due to the decline in earnings per share. Therefore, a reasonable performance goal is necessary for venture capital institutions to obtain considerable financial returns and for entrepreneurs to win the right of control.

### The general structure of the VAM agreement

Through the analysis of Mengniu’s and Yongle’s cases, we propose a general structure of the VAM agreement as follows:

Enterprise profit is the trigger condition of the agreement’s implementation, and equity is the adjustment object of the deal. The initial profit of the startup when it obtains the investment is *π*_0_, the proportion of equity obtained by venture capital institutions is *S* (0<*S*<1). The agreement duration is T, and the performance target is π*.Both parties agree that if *π*_*t*_<π* within the duration of the agreement, the entrepreneur will transfer *δ*_1_ shares to the venture capital institutions. Conversely, the venture capital institutions transfer *δ*_2_ shares to the entrepreneur.

The structure takes profit as the indicator of performance evaluation. It reflects that most agreements take simple and intuitive financial indicators as trigger conditions. In contrast, non-financial performance indicators or a combination of the two are rarely seen, and the profit level can most directly reveal the enterprise’s business performance. Therefore, according to the VAM agreement of Yongle, the enterprise profit is the performance target for the valuation adjustment of both parties.

The structure takes equity as the object of valuation adjustments. Venture capital institutions are financial investors who pursue the maximization of interests. The equity reflects the overall value of the enterprise, so investors usually take the equity of the financing party as the bargaining chip of the VAM agreement to realize a change of a certain number of shares and even transfer of controlling rights according to the trigger conditions.

The structure reflects entrepreneurs’ incentive and restraint mechanism of equity incentive and punishment. In the VAM agreement of Mengniu’s second-round financing, it is agreed that if Mengniu achieves the performance target, it can get an equity reward. Otherwise, it must transfer equal shares to the investor as punishment. Without losing generality, it is assumed that transferred shares are not necessarily identical, but *δ*_1_ and *δ*_2_ shares are respectively transferred to the other party based on whether the performance target is reached. In Mengniu’s first round of financing, the VAM agreement only stipulates incentives with no punitive measures, which is equivalent to *δ*_1_ = 0.

The structure allows valuation adjustments to be performed in advance. The VAM agreement stipulates that the performance of the enterprise shall be inspected during the agreement period. If the performance increases significantly during the agreement period and meets the trigger conditions, the equity delivery shall be carried out in advance; otherwise, the delivery will be carried out at the end of the agreement period. If the agreement period is extended, it can wait for the achievement of the performance goal of the startup for a longer time.

**Fig 3:**
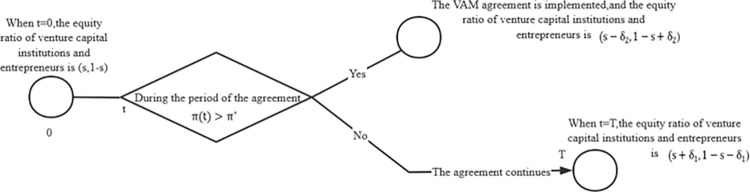
General structure and execution sequence of the VAM agreement.

The general structure and execution sequence of the VAM agreement is shown in Fig 3.

### Value process description of startup

Uncertainty about development prospects is a significant feature of startups. Both sides of investment and financing are faced with the uncertainty of future profit levels when signing the VAM agreement. Considering the impact of a series of uncertain factors such as technology and market demand on the operating performance of a startup, it is assumed that the profit flow *π*_*t*_ follows the stochastic process described by geometric brownian motion,

$$d\pi_{t}={\mu \pi }_{t}dt+\sigma \pi_{t}dW_{t}$$ (1)

Where *μ* is the drift term, which represents the expected growth rate of enterprise profits; *σ* is the volatility item, which indicates the risk level of enterprise profit; *dW*_*t*_ is the increment of the Wiener process. It is assumed that the expected growth rate of corporate profits is mainly determined by the entrepreneurial effort $\mu \in (\underline{\mu },\bar{\mu })$ after the VAM agreement is reached.

The uncertainty of profit also brings the uncertainty of the startup’s value. Given the profit *π*_*t*_ at time *t*, then the conditional expectation of profit at time s is $E(\pi_{s}|\pi_{t})]=\pi_{t}e^{\mu (s-t)}$. The venture capital institutions price the future expected profit flow with the rate of return *R*. Thus, the project value is the expected present value of the future profit flow, and the project value *V*_*t*_ at time *t* is:

$$V_{t}=E\left[\int_{t}^{\infty }\pi_{s}e^{-R(s-t)}ds|\pi_{t}\right]=\int_{t}^{\infty }\pi_{t}e^{-(R-\mu )(s-t)}ds=\frac{\pi_{t}}{R-\mu }$$ (2)

According to Ito’s lemma,

$$dV_{t}(\pi_{t})=V_{t}^{\prime }\;d\pi_{t}+\frac{1}{2}V_{t}^{\prime \prime }\;{\left(d\pi_{t}\right)}^{2}=\frac{d\pi_{t}}{R-\mu }=\frac{\pi_{t}}{R-\mu }(\mu dt+\sigma dW_{t})$$ (3)

Therefore, the evolution of project value *V*_*t*_ follows the same geometric brownian motion.


$$dV_{t}=\mu V_{t}dt+\sigma V_{t}dW_{t}$$ (4)

The assumption of startups’ profit and value process in this paper is the same as Hsu’s (2010) [36], and geometric brownian motion describes the uncertainty of entrepreneurial value. We further considered the issue of the moral hazard of startups by assuming that the effort level of entrepreneurs determines the expected growth rate of the startup’s value. In this way, we can consider uncertainty characteristics and behavioral choices for startups in analyzing optimal performance targets.

### The payoff of venture capital institutions with the VAM agreement

#### The option value of the VAM agreement

VAM agreements give venture capital institutions the contractual right to adjust valuations so that when the deal ends, the venture capital institutions have two kinds of payoff outcomes based on whether the pre-agreed performance targets are met. One scenario is when performance targets are achieved, where venture capital institutions need to transfer part of their stake to the financier, but the startup has a higher valuation. Another scenario is when performance targets are not met, and equity compensation can be obtained from the financier, but the startup’s valuation declines. Given the structure of the VAM agreement and preset performance target, at the beginning of the agreement, the option value held by the venture capital institutions is the present value of the contingent payoff at the end of the agreement period. Therefore, we first derive the option value held by the venture capital institutions under the VAM agreement and put forward the following proposition, which is the basis for the decision-making of the venture capital institutions.

**Proposition 1: The option value of the VAM agreement is**,

$$\Phi (\pi_{0})=(s+\delta_{1})\frac{\pi_{0}}{R-\mu }+(\delta_{1}+\delta_{2})\frac{\pi_{0}}{R-\mu }{N(d}_{1})-(\delta_{1}+\delta_{2})\frac{\pi_{0}}{R-\mu }{\left(\frac{\pi^{*}}{\pi_{0}}\right)}^{\frac{2r}{\sigma^{2}}+1}N(-d_{2})$$ (5)

Where,

$$d_{1}=\frac{1}{\sigma \sqrt{T}}[\log\left(\frac{\pi^{*}}{\pi_{0}}\right)-(r+\frac{1}{2}\sigma^{2})T],$$

$d_{2}=\frac{1}{\sigma \sqrt{T}}[\log\left(\frac{\pi^{*}}{\pi_{0}}\right)+(r+\frac{1}{2}\sigma^{2})T]$, ℕ(·) is the standard normal distribution function.

The proof is as follows:

At the end of the VAM agreement (*T*), the value of a startup (*V*_*T*_) is $\frac{\pi_{T}}{R-\mu }$. According to the VAM agreement, if the profit of the startup does not reach π* during the agreement period, the payoff of the venture capital institutions at the end of agreement period is (*s*+*δ*_1_)*V*_*T*_, if the profit of the startup reaches π* during the agreement period, the payoff of the venture capital institutions at the end of agreement period is (*s*−*δ*_2_)*V*_*T*_.

Let $\tilde{W_{t}}(0\leq t\leq T)$ be brownian motion under the risk-neutral measure $\tilde{P}$, then the profit flow of the startup is subject to $d\pi_{t}=r\pi_{t}dt+\sigma \pi_{t}d\tilde{W_{t}}$, where *π*_*t*_ is written as $\pi_{t}=\pi_{0}e^{\sigma \tilde{W_{t}}+(r-\frac{1}{2}\sigma^{2})t}$. Let $\hat{W_{t}}=\alpha t+\tilde{W_{t}},\alpha =\frac{1}{\sigma }(r-\frac{1}{2}\sigma^{2})$, then $\pi_{t}=\pi_{0}e^{\sigma \hat{W_{t}}}$, define $\hat{M_{T}}=\underset{0\leq t\leq T}{\max}\hat{W_{t}}$, the maximum profit of the venture project during the agreement period $\underset{0\leq t\leq T}{\max}\pi_{t}=\pi_{0}e^{\sigma \hat{M_{T}}}$.

The payoff outcome of the venture capital institutions at the end of the agreement period can be expressed as follows:

$$\Phi (\pi_{T})=(s+\delta_{1})\frac{\pi_{T}}{R-\mu }I\{\pi_{0}e^{\sigma \hat{M_{T}}}<\pi^{*}\}+(s-\delta_{2})\frac{\pi_{T}}{R-\mu }I\{\pi_{0}e^{\sigma \hat{M_{T}}}\geq \pi^{*}\}$$ (6)

At the time of signing the contract, the value of the contractual option owned by the venture capital institutions is,

$$\begin{array}{l} \Phi (\pi_{0})=\tilde{E}[e^{-rT}\Phi (\pi_{T})]\\ =\frac{1}{R-\mu }\tilde{E}\left[e^{-rT}(s+\delta_{1})\pi_{T}I\{\pi_{0}e^{\sigma \hat{M_{T}}}<\pi^{*}\}\right]+\frac{1}{R-\mu }\tilde{E}\left[e^{-rT}(s-\delta_{2})\pi_{T}I\{\pi_{0}e^{\sigma \hat{M_{T}}}\geq \pi^{*}\}\right]\\ ≝\Phi_{1}(\pi_{0})+\Phi_{2}(\pi_{0}) \end{array}$$ (7)

Under $\tilde{P}$, the joint density of ($\hat{M_{T}},\hat{W_{T}}$) is ${\tilde{f}}_{\hat{M_{T}},\hat{W_{T}}}(m,w),{\tilde{f}}_{\hat{M_{T}},\hat{W_{T}}}(m,w)=\frac{2(2m-w)}{t\sqrt{2\pi t}}e^{\alpha w-{\frac{1}{2}\alpha }^{2}T-\frac{1}{2T}{\left(2m-w\right)}^{2}},$ *w*≤*m*, *m*>0, let $\pi =\frac{1}{\sigma }log\frac{\pi^{*}}{\pi_{0}}$.


$$\begin{array}{l} \Phi_{1}(\pi_{0})=\frac{1}{R-\mu }[\int_{-\infty }^{0}\int_{0}^{\pi }+\int_{0}^{\pi }\int_{w}^{\pi }]e^{-rT}{(s+\delta_{1})\pi }_{0}e^{\sigma w}\frac{2(2m-w)}{T\sqrt{2\pi T}}e^{\alpha w-{\frac{1}{2}\alpha }^{2}T-\frac{1}{2T}{\left(2m-w\right)}^{2}}dmdw\\ \;=\frac{1}{R-\mu }\int_{-\infty }^{\pi }\int_{w^{+}}^{\pi }e^{-rT}{(s+\delta_{1})\pi }_{0}e^{\sigma w}\frac{2(2m-w)}{T\sqrt{2\pi T}}e^{\alpha w-{\frac{1}{2}\alpha }^{2}T-\frac{1}{2T}{\left(2m-w\right)}^{2}}dmdw\\ \;=-\frac{1}{R-\mu }\int_{-\infty }^{\pi }e^{-rT}{{(s+\delta_{1})\pi }_{0}}_{0}e^{\sigma w}\frac{1}{\sqrt{2\pi T}}e^{\alpha w-{\frac{1}{2}\alpha }^{2}T-\frac{1}{2T}{\left(2m-w\right)}^{2}}{|}_{m=w^{+}}^{m=\pi }dw\\ \;={\frac{1}{R-\mu }{(s+\delta_{1})\pi }_{0}}_{0}I_{1}-\frac{1}{R-\mu }{(s+\delta_{1})\pi }_{0}I_{2} \end{array}$$ (8)

Where,

$$I_{1}=\frac{1}{\sqrt{2\pi T}}\int_{-\infty }^{\pi }e^{\sigma w-rT+\alpha w-{\frac{1}{2}\alpha }^{2}T-\frac{1}{2T}w^{2}}dw,I_{2}=\frac{1}{\sqrt{2\pi T}}\int_{-\infty }^{\pi }e^{\sigma w-rT+\alpha w-{\frac{1}{2}\alpha }^{2}T-\frac{1}{2T}{\left(2\pi -w\right)}^{2}}dw$$ (9)

$$I_{1}=e^{\frac{1}{2}{\left(\alpha +\sigma \right)}^{2}T-rT-\frac{1}{2}\alpha^{2}T}\frac{1}{\sqrt{2\pi }}\int_{-\infty }^{\frac{\pi -(\alpha +\sigma )T}{\sqrt{T}}}e^{-\frac{1}{2}{\left[\frac{w-(\alpha +\sigma )T}{\sqrt{T}}\right]}^{2}}d[\frac{w-(\alpha +\sigma )T}{\sqrt{T}}],$$

by substituting $\alpha =\frac{1}{\sigma }(r-\frac{1}{2}\sigma^{2})$ into Formula (9).

$I_{1}=N\left(\frac{1}{\sigma \sqrt{T}}[\log\left(\frac{\pi^{*}}{\pi_{0}}\right)-(r+\frac{1}{2}\sigma^{2})T]\right)≝N(d_{1})$, ℕ(·) is the standard normal distribution function.

$$\begin{array}{l} I_{2}=e^{\frac{1}{2}{\left(\alpha +\sigma +\frac{2\pi }{T}\right)}^{2}T-rT-{\frac{1}{2}\alpha }^{2}T-\frac{2\pi^{2}}{T}}\frac{1}{\sqrt{2\pi }}\int_{-\infty }^{\frac{\pi -(\alpha +\sigma +\frac{2\pi }{T})T}{\sqrt{T}}}e^{-\frac{1}{2}{\left[\frac{w-(\alpha +\sigma +\frac{2\pi }{T})T}{\sqrt{T}}\right]}^{2}}d\left[\frac{w-(\alpha +\sigma +\frac{2\pi }{T})T}{\sqrt{T}}\right]\\ ={\left(\frac{\pi^{*}}{\pi_{0}}\right)}^{\frac{2r}{\sigma^{2}}+1}N\left(-\frac{1}{\sigma \sqrt{T}}[\log\left(\frac{\pi^{*}}{\pi_{0}}\right)+(r+\frac{1}{2}\sigma^{2})T]\right)\\ ≝{\left(\frac{\pi^{*}}{\pi_{0}}\right)}^{\frac{2r}{\sigma^{2}}+1}N(-d_{2}) \end{array}$$ (10)

Similarly,

$$\begin{array}{l} \Phi_{2}(\pi_{0})=\frac{1}{R-\mu }[\int_{-\infty }^{\pi }\int_{\pi }^{+\infty }+\int_{\pi }^{+\infty }\int_{w}^{+\infty }]e^{-rT}{s\pi }_{0}e^{\sigma w}\frac{2(2m-w)}{T\sqrt{2\pi T}}e^{\alpha w-{\frac{1}{2}\alpha }^{2}T-\frac{1}{2T}{\left(2m-w\right)}^{2}}dmdw\\ \;=\frac{1}{R-\mu }{(s-\delta_{2})\pi }_{0}N({-d}_{1})+\frac{1}{R-\mu }{(s-\delta_{2})\pi }_{0}{\left(\frac{\pi^{*}}{\pi_{0}}\right)}^{\frac{2r}{\sigma^{2}}+1}N(-d_{2}) \end{array}$$ (11)

The option value of the VAM agreement is:

$$\begin{array}{l} \Phi (\pi_{0})=\Phi_{1}(\pi_{0})+\Phi_{2}(\pi_{0})\\ \;=(s+\delta_{1})\frac{\pi_{0}}{R-\mu }+(\delta_{1}+\delta_{2})\frac{\pi_{0}}{R-\mu }{N(d}_{1})-(\delta_{1}+\delta_{2})\frac{\pi_{0}}{R-\mu }{\left(\frac{\pi^{*}}{\pi_{0}}\right)}^{\frac{2r}{\sigma^{2}}+1}N(-d_{2}) \end{array}$$ (12)

### The effect of performance target on the option value

Venture capital institutions maximize the value of agreements they hold by negotiating or dominating the core terms of performance targets. Therefore, we further examine the impact of performance targets on the agreement’s value and propose the following proposition.

**Proposition 2: The higher the performance target, the greater the option value held by the venture capital institutions.**

The proof is as follows:

Under different performance targets, we compare the payoff of venture capital institutions and suppose *π*_1_*>*π*_2_*,

When π* = *π*_1_*, the payoff of the venture capital institutions at the end of the agreement period can be expressed as:

$$\Phi (\pi_{T},\;M_{T};{\pi_{1}}^{*})=(s+\delta_{1})\frac{\pi_{T}}{R-\mu }I\{\pi_{0}e^{\sigma \hat{M_{T}}}<{\pi_{1}}^{*}\}+(s-\delta_{2})\frac{\pi_{T}}{R-\mu }I\{\pi_{0}e^{\sigma \hat{M_{T}}}\geq {\pi_{1}}^{*}\}$$ (13)

When π* = *π*_2_*, the payoff of the venture capital institutions at the end of the agreement period can be expressed as:

$$\Phi (\pi_{T},\;M_{T};\;{\pi_{2}}^{*})=(s+\delta_{1})\frac{\pi_{T}}{R-\mu }I\{\pi_{0}e^{\sigma \hat{M_{T}}}<{\pi_{2}}^{*}\}+(s-\delta_{2})\frac{\pi_{T}}{R-\mu }I\{\pi_{0}e^{\sigma \hat{M_{T}}}\geq {\pi_{2}}^{*}\}$$ (14)

Thus,

$$\Phi (\pi_{T},\;M_{T};\;{\pi_{1}}^{*})-\Phi (\pi_{T},\;M_{T};\;{\pi_{2}}^{*})=(\delta_{1}+\delta_{2})\frac{\pi_{T}}{R-\mu }I\{{\pi_{2}}^{*}\leq \pi_{0}e^{\sigma \hat{M_{T}}}<{\pi_{1}}^{*}\}\equiv X\geq 0$$ (15)

$$And,\Phi (\pi_{0},\;\pi^{*})=\tilde{E}[e^{-rT}\Phi (\pi_{T})]$$ (16)

According to the nature of expectations, *Φ*(*π*_0_; *π*_1_*)≥*Φ*(*π*_0_; *π*_2_*).

Inequality is strictly true in consideration of the following equation,

$$\Phi (\pi_{0};\;{\pi_{1}}^{*})=\Phi (\pi_{0};\;{\pi_{2}}^{*})+\tilde{E}[e^{-rT}X]$$ (17)

And $\tilde{E}[e^{-rT}X]=e^{-rT}\tilde{E}[X]>0$, Therefore, the inequality sign is strictly established.

Proposition 2 proves that under the contract structure of the VAM agreement, the higher the performance target set by the venture capital institutions, the greater the option value of the agreement. An intuitive reason is that venture capital institutions benefit from rising share values when the performance target is met and benefit from compensation when performance declines, which locks in the risk to a certain extent. Therefore, venture capital institutions tend to set high-performance targets if they only consider the option value of the VAM agreement and do not consider whether the performance can be achieved.

**Fig 4:**
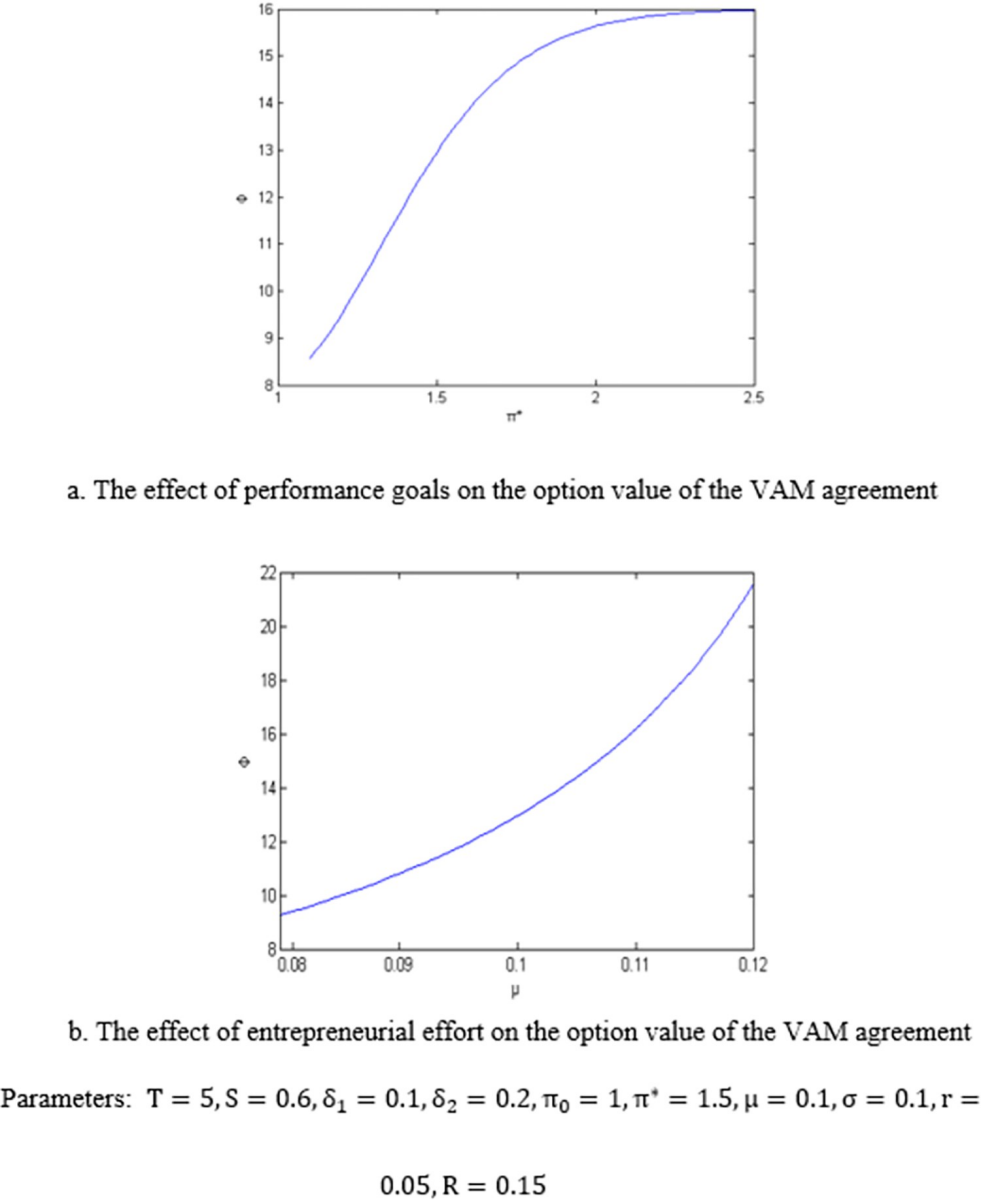
The option value of the VAM agreement.

Based on the analytic solution of the option value of the VAM agreement obtained from Proposition 1, the effect of performance target and entrepreneurial effort on the option value of the VAM agreement are numerically illustrated in Fig 4. It can be seen that the option value of the VAM agreement is an increasing function of the performance target and the entrepreneur’s effort.


$$Parameters:\;T=5,S=0.6,{\delta_{1}=0.1,\delta }_{2}=0.2,\pi_{0}=1,\pi^{*}=1.5,\mu =0.1,\sigma =0.1,r=0.05,R=0.15$$

### Optimal performance target of the VAM agreement

#### The behavior choice of entrepreneurs

As financial investors, venture capital institutions only focus on the monetary return and are value maximizers. Different from venture capital institutions, entrepreneurs do not simply regard venture projects as ordinary investments. Besides paying attention to financial returns, they also emphasize corporate control. Hsu (2010) [36] argues that objectively estimating the value obtained by entrepreneurs in project operations is difficult, so it is assumed that entrepreneurs are probability-maximizers. From this point of view, this paper argues entrepreneurs have a strong desire to achieve performance goals during the agreement period. This is because if the performance reaches the trigger conditions specified in the agreement, the entrepreneurs will get share rewards, strengthen the control of the enterprise, and also get the favor of other investors. Therefore, the expected time to achieve the performance goal π* is taken as the optimization goal of entrepreneurs. After the agreement is reached, the entrepreneur chooses the effort degree *μ* to minimize the expected time to achieve the performance goal π* in the agreement period. Under this assumption, the following propositions are proved:

**Proposition 3: Once the VAM agreement concludes, the entrepreneur chooses the highest level of effort.**

The proof is as follows:

By Ito’s lemma,

$$d(ln\pi_{t})=\frac{\partial (ln\pi_{t})}{\partial t}dt+\frac{\partial (ln\pi_{t})}{\partial \pi_{t}}d\pi_{t}+\frac{1}{2}\frac{\partial^{2}(ln\pi_{t})}{\partial {\left(\pi_{t}\right)}^{2}}{\left(d\pi_{t}\right)}^{2}=\frac{1}{\pi_{t}}d\pi_{t}-\frac{1}{2}\frac{{\left(d\pi_{t}\right)}^{2}}{{\pi_{t}}^{2}}$$ (18)

The result of $d(ln\pi_{t})=(\mu -{\frac{1}{2}\sigma }^{2})dt+\sigma dW_{t}$ is obtained by substituting $\frac{d\pi_{t}}{\pi_{t}}=\mu dt+\sigma dW_{t}$, that is, lnπ_t_ obeys brownian motion with drift rate $\mu -{\frac{1}{2}\sigma }^{2}$ and volatility rate *σ*.

Let *T*_π*_ be the time when the profit level reaches the performance target π*, $T_{\pi^{*}}=in\;f\left\{t|\pi_{t}\geq \pi^{*}\right\}$, the initial value of Brownian motion is *lnπ*_0_, the probability density function of *T*_π*_ is,

$$f(T_{\pi^{*}};ln\pi_{0},ln\pi^{*})=\frac{ln\pi^{*}-ln\pi_{0}}{\sigma \sqrt{2\pi {T_{\pi^{*}}}^{3}}}exp\{-\frac{{\left[ln\pi^{*}-ln\pi_{0}-(\mu -{\frac{1}{2}\sigma }^{2})T_{\pi^{*}}\right]}^{2}}{2\sigma^{2}T_{\pi^{*}}}\}$$ (19)

And the Laplace transformation of *T*_π*_ is,

$$E(e^{-\theta T_{\pi^{*}}})=\int_{0}^{\infty }e^{-\theta T_{\pi^{*}}}f(T_{\pi^{*}})dT_{\pi^{*}}=exp\{-\frac{\ln\left(\pi^{*}/\pi_{0}\right)}{\sigma^{2}}[\sqrt{{\left(\mu -{\frac{1}{2}\sigma }^{2}\right)}^{2}+{2\sigma }^{2}\theta }-(\mu -{\frac{1}{2}\sigma }^{2})]\}$$ (20)

It can be inferred that the expected time for the profit level to reach the performance target π* is,

$$E(T_{\pi^{*}})=\int_{0}^{\infty }T_{\pi^{*}}f(T_{\pi^{*}})dT_{\pi^{*}}=-\underset{\theta \to 0}{\lim}\frac{\partial E(e^{-\theta T_{\pi^{*}}})}{\partial \theta }=\frac{\ln\left(\pi^{*}/\pi_{0}\right)}{\mu -{\frac{1}{2}\sigma }^{2}}$$ (21)

For a given π*, entrepreneurs choose effort *μ* to minimize the expected time to reach the preset target π*, and differentiates $E(T_{\pi^{*}})$ to get the result,

$$\frac{\partial E(T_{\pi^{*}})}{\partial \mu }=-\frac{\ln\left(\pi^{*}/\pi_{0}\right)}{{\left(\mu -{1/2\sigma }^{2}\right)}^{2}}<0$$ (22)

For entrepreneurs, the expected time to achieve the performance target is a monotonic decreasing function of the level of effort. Therefore, after the VAM agreement concludes, entrepreneurs will choose the highest level of effort.

**Fig 5:**
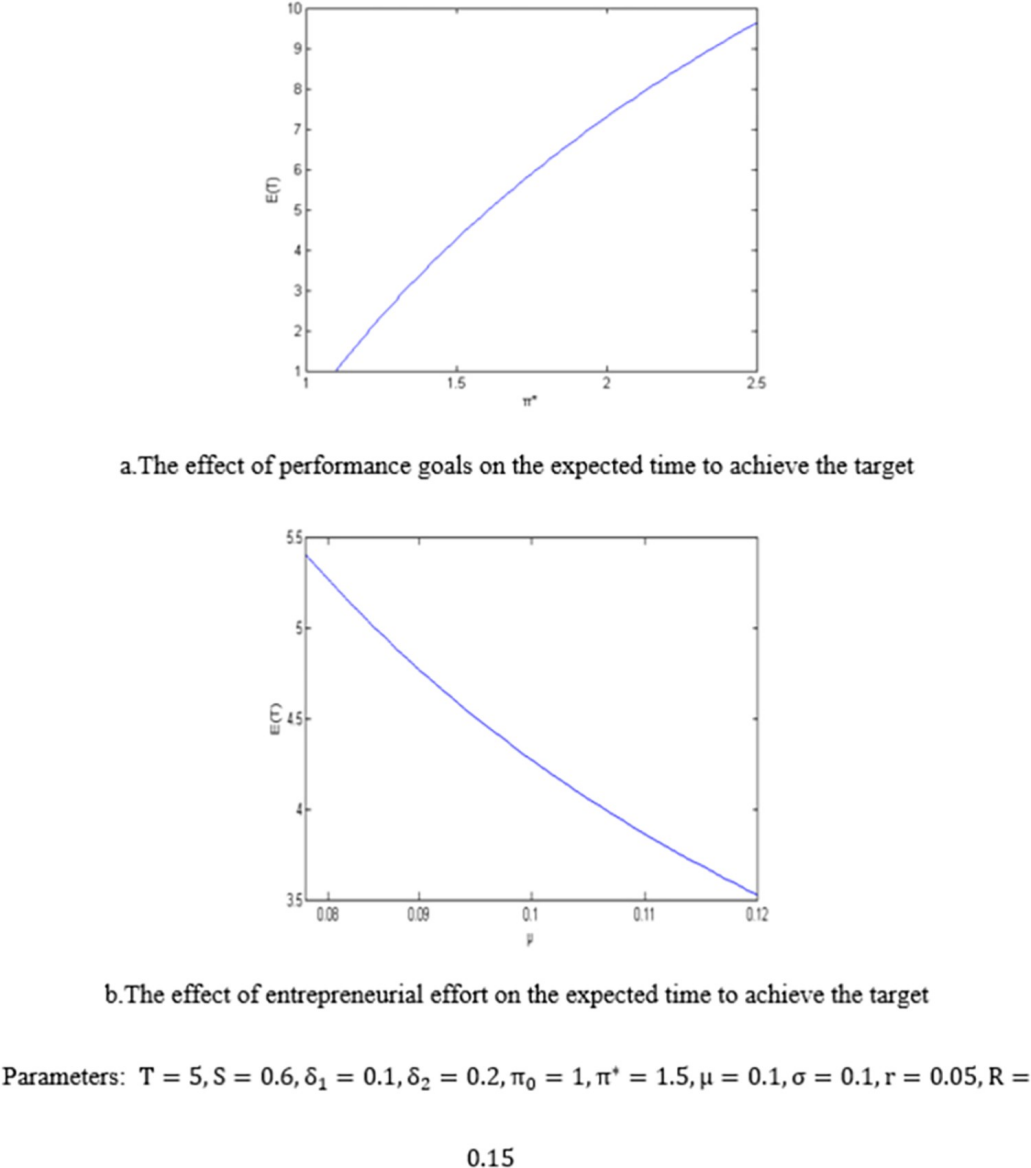
The expected time to achieve the performance target.

Based on the analytic solution of the expected time for entrepreneurs to achieve the performance target obtained in proposition 3, the effect of the performance target and entrepreneurial effort on the expected time to complete the target is numerically illustrated in Fig 5.


$$Parameters:\;T=5,S=0.6,{\delta_{1}=0.1,\delta }_{2}=0.2,\pi_{0}=1,\pi^{*}=1.5,\mu =0.1,\sigma =0.1,r=0.05,R=0.15$$

It can be seen that the higher the performance goal setting, the longer the expected time to achieve the goal, while the higher the degree of effort of entrepreneurs, the shorter the expected time to complete the performance goal. Proposition 3 shows the VAM agreement can effectively encourage entrepreneurs to make the best efforts to maximize the interests of venture capital institutions.

### The optimal performance target setting

Considering only the option value of the VAM agreement, it seems that venture capital institutions should set high-performance targets. But it is evident that performance targets π* cannot be increased indefinitely. Considering the participation constraints of entrepreneurs, that is after the entrepreneurs do their best $\bar{\mu }$, the expected time to achieve a performance goal π* can not be greater than the agreement period, and thus π* has an optimal upper limit, which is given by proposition 4.

**Proposition 4:Assuming that venture capital institutions maximize their economic value with the participation constraints of entrepreneurs, the optimal performance target is set as**,

$$\pi_{optimal}^{*}=\pi_{0}exp[(\bar{\mu }-\frac{1}{2}\sigma^{2})T]$$ (23)

The proof is as follows:

From proposition 3, once the performance target π* is given, it is known that the entrepreneur will choose the highest level of effort $\bar{\mu }$, and the expected time to achieve the performance target is,

$${E(T_{\pi^{*}})}_{min}=\frac{\ln\left(\pi^{*}/\pi_{0}\right)}{\bar{\mu }-\frac{1}{2}\sigma^{2}}$$ (24)

To meet the participation constraints of entrepreneurs, that is, to achieve the performance goals in the agreement period, there is $minE(T_{\pi^{*}})\leq T$. The performance target must satisfy $\pi^{*}\leq \pi_{0}exp[(\bar{\mu }-1/2\sigma^{2})T]$. From proposition 2, the higher the performance target set in the VAM agreement, the higher the option value of the agreement owned by the venture capital institutions. Therefore, the optimal performance target of the venture capital institutions is set as the upper limit,

$$\pi_{optimal}^{*}=\pi_{0}exp[(\bar{\mu }-1/2\sigma^{2})T]$$ (25)

Faced with the uncertainty of the business environment, rational entrepreneurs can only accept the performance goals that can be expected to be achieved within the agreement period. Therefore, to meet the participation constraints of entrepreneurs, venture capital institutions will set high-performance targets as far as possible to obtain maximum payoff under the contractual structure of the VAM agreements.

Differentiates the agreement period *T* and the risk level *σ* as follows,

$$\frac{\partial \pi_{optimal}^{*}}{\partial T}=\pi_{0}\exp\left[(\bar{\mu }-\frac{1}{2}\sigma^{2})T\right]\left(\bar{\mu }-\frac{1}{2}\sigma^{2}\right)>0$$ (26)

$$\frac{\partial \pi_{optimal}^{*}}{\partial \sigma }=-\pi_{0}\exp\left[(\bar{\mu }-\frac{1}{2}\sigma^{2})T\right]\sigma <0$$ (27)

It can be found that the optimal performance target setting should be an increasing function of the duration of the agreement, which means that if the period of the agreement is short, the performance target requirements should be reduced. On the other hand, if the performance target setting is high, the duration of the contract should be appropriately extended to expect the enterprise to operate rationally. In addition, the optimal performance target setting should be a decreasing function of the enterprise risk level, which means that the performance target requirements should be reduced in the face of high business risk.

### Limitations and future research direction

This paper extracts the general structure of the VAM agreement through a typical case analysis. It gives the optimal pre-determined profit target, contingent on which the venture capital institutions exercise a contractual right to adjust the startup’s valuation. The findings provide essential insight and principles for setting performance goals in VAM agreements. For example, venture capital institutions firms should not over-exploit negotiation advantages to set excessively high-performance targets so that entrepreneurs have reasonable expectations for achieving performance goals with the best efforts. Moreover, under the assumption of a continuous stochastic process of project value, we can obtain an accurate mathematical expression of the optimal performance target. However, the specific performance target clauses of VAM agreements vary widely. In addition to the most commonly used financial indicators, such as profits, revenue, etc., some non-financial indicators, such as the acquisition of patents, the retention rate of core scientific and technological personnel, and the industrialization of new technologies, will also be used as ‘bet-on’ targets. In this case, the principles we advocate for setting optimal performance goals may still apply. Still, the uncertain portrayal of non-financial indicators will be more complex, such as a random jump process, making it difficult to obtain a concise mathematical expression of the optimal performance target. In future research, it is an important research direction to adopt a more comprehensive approach to study the performance target setting of non-financial indicators in VAM agreements to more fully reflect practical operations.

## Conclusion

The VAM agreement is a prevalent investment tool in emerging markets with the agreement upon fulfilling a particular performance target, contingent on which the investor may exercise a contractual right to adjust the valuation by altering the relative shareholding or other financial positions. Motivated by exploring reasonable performance targets to improve the effectiveness of the VAM agreement and achieve a win-win situation for both investment and financing parties, this paper extracts the general framework of the VAM agreement through typical case studies and then exploits the real options method first to derive the impact of the performance target on the option value held by venture capital institutions, and then analyze the behavior choices and participation constraints of entrepreneurs under the VAM agreement. Assuming that the venture capital institutions maximize the value of the agreement they hold under the condition of the participation of entrepreneurs, that is, entrepreneurs have reasonable expectations for the achievement of performance goals, the paper derives the mathematical formula of optimal performance target. From the perspective of venture capital institutions, we derive the option value of the VAM agreement and prove that under the structure of the VAM agreement, the payoff of the venture capital institutions is the increased function of the preset performance target. This may explain that venture capital institutions tend to set higher performance targets from the perspective of real options, especially when they have a strong negotiating advantage. From a startup’s perspective, we derive the expected time to achieve performance goals under uncertain conditions and prove that the expected time is a subtractive function of the entrepreneur’s effort and an increased function of the performance target. This result confirms the incentive effect of the VAM agreements on entrepreneurs, i.e., entrepreneurs will make the most effort to achieve their performance goals. More importantly, this result provides a constraint to the search for rational performance goal setting, i.e., the expected time to complete the performance goal with the best effort of the entrepreneur should be at least within the agreement period. Thus, we derive an optimal performance goal setting, which is the increasing function of the agreement period and the subtraction function of project risk. To achieve a win-win situation, venture capital institutions should fully consider the participation constraints of entrepreneurs and the internal and external risks of the projects other than set excessively high-performance targets, which may lead to irrational speculation and reduce the effectiveness of VAM agreements.

## Supporting information

S1 Fig Mengniu’s VAM agreement. (TIF)

S2 Fig Yongle’s VAM agreement. (TIF)

S3 Fig General structure and execution sequence of the VAM agreement. (TIF)

S4 Fig The option value of the VAM agreement. (TIF)

S5 Fig The expected time to achieve the performance target. (TIF)

S1 Text Parameters. (DOCX)
