# Supplementary figures and images for: The optimal performance target of valuation adjustment mechanism agreement with real options perspective
Source: PLoS One. 2022 Nov 21;17(11):e0277509. doi: 10.1371/journal.pone.0277509 (PMC9678305; doi:10.1371/journal.pone.0277509)

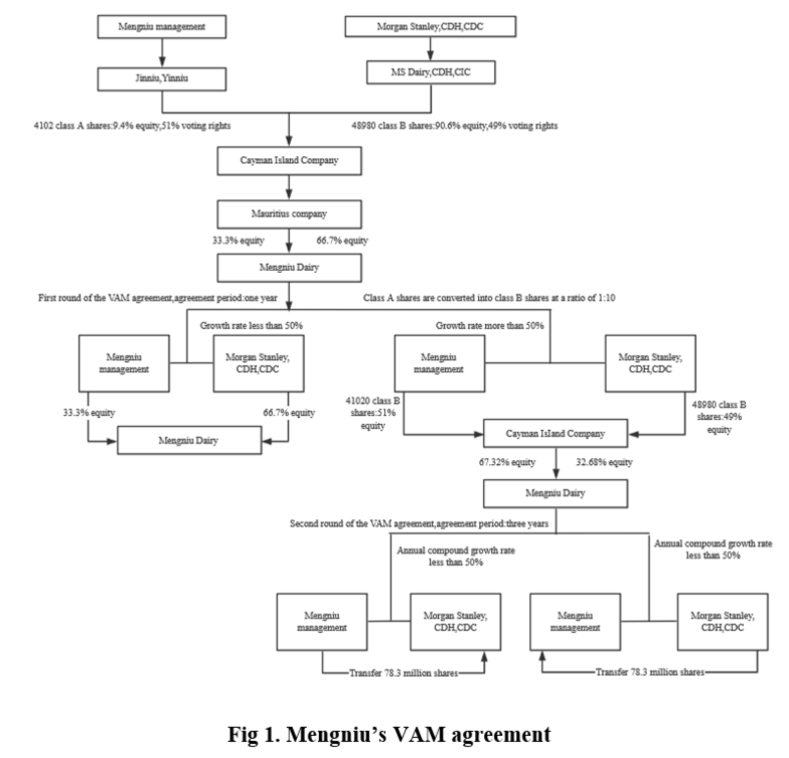

Supplement: S1 Fig — (TIF) [file pone.0277509.s001.tif]

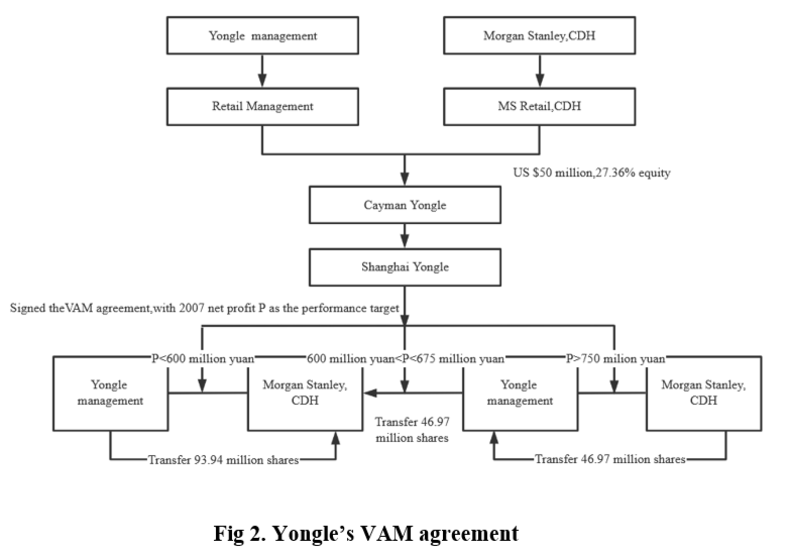

Supplement: S2 Fig — (TIF) [file pone.0277509.s002.tif]

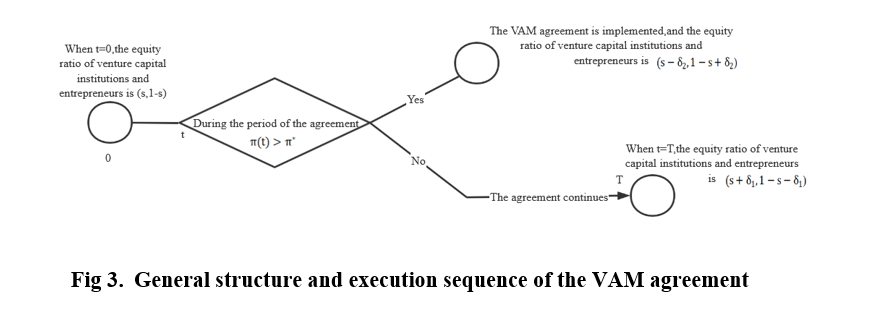

Supplement: S3 Fig — (TIF) [file pone.0277509.s003.tif]

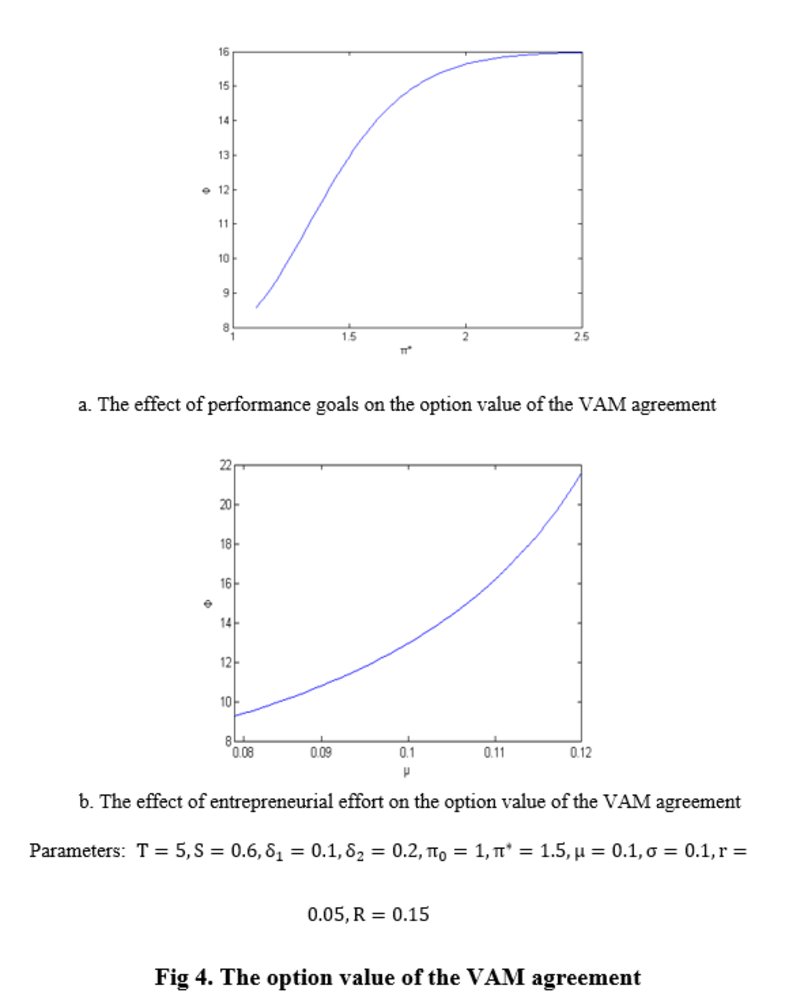

Supplement: S4 Fig — (TIF) [file pone.0277509.s004.tif]

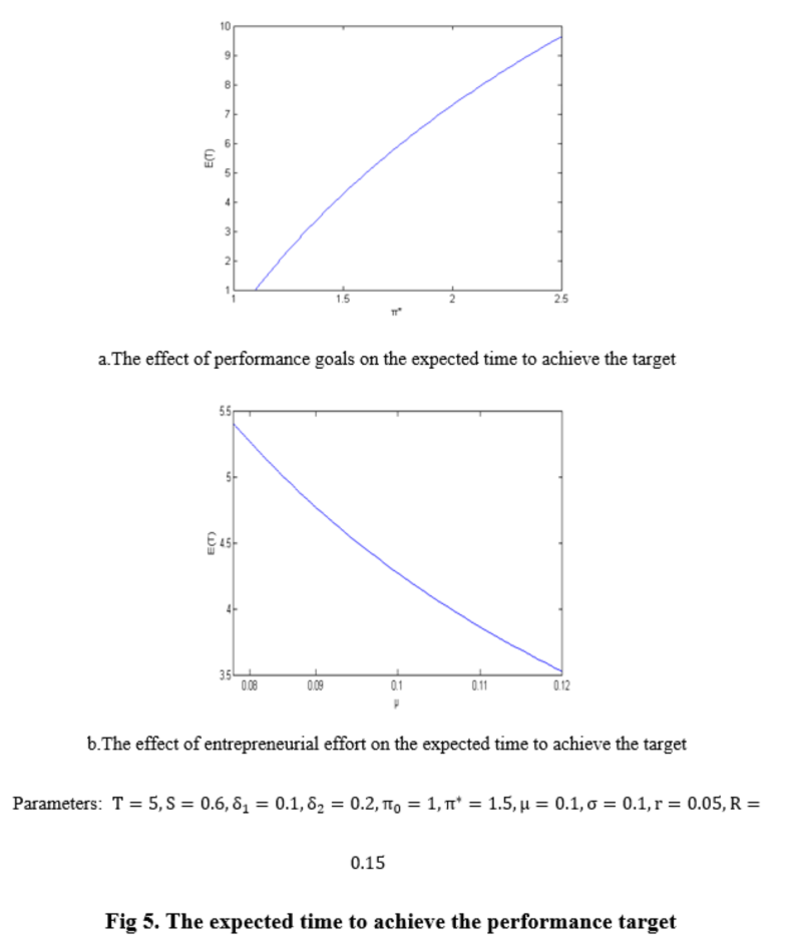

Supplement: S5 Fig — (TIF) [file pone.0277509.s005.tif]
