# Supplementary material for: The optimal performance target of valuation adjustment mechanism agreement with real options perspective
Source: PLoS One. 2022 Nov 21;17(11):e0277509. doi: 10.1371/journal.pone.0277509 (PMC9678305; doi:10.1371/journal.pone.0277509)
Supplement: S1 Text — (DOCX) [file pone.0277509.s006.docx]

The dataset (parameters) for reproducing the results visualized in Fig 4 is: $T=5,S=0.6, {\delta_{1}=0.1,\delta}_{2}=0.2, \pi_{0}=1,\pi^{*}=1.5, \mu=0.1,\sigma=0.1, r=0.05,R=0.15$.

The dataset (parameters) for reproducing the results visualized in Fig 5 is:$T=5,S=0.6, {\delta_{1}=0.1,\delta}_{2}=0.2, \pi_{0}=1,\pi^{*}=1.5, \mu=0.1,\sigma=0.1, r=0.05,R=0.15$.

The parameters are also listed below each figure in the manuscript for easy reference.
